# Deaths Attributable to Carbapenem-Resistant *Enterobacteriaceae* Infections

**DOI:** 10.3201/eid2007.121004

**Published:** 2014-07

**Authors:** Matthew E. Falagas, Giannoula S. Tansarli, Drosos E. Karageorgopoulos, Konstantinos Z. Vardakas

**Affiliations:** Alfa Institute of Biomedical Sciences, Athens, Greece (M.E. Falagas, G.S. Tansarli, D.E. Karageorgopoulos, K.Z. Vardakas);; Tufts University School of Medicine, Boston, Massachusetts, USA (M.E. Falagas)

**Keywords:** carbapenem resistance, bloodstream infection, Enterobacteriaceae, carbapenem-resistant Enterobacteriaceae, infections, Klebsiella pneumoniae, Klebsiella pneumoniae carbapenemase, KPC, bacteremia, carbapenemases, carbapenemase-producing, attributable mortality, mortality, attributable deaths, deaths, systematic review, metaanalysis, meta-analysis, outcome, bacteria

## Abstract

We evaluated the number of deaths attributable to carbapenem-resistant *Enterobacteriaceae* by using studies from around the world published before April 9, 2012. Attributable death was defined as the difference in all-cause deaths between patients with carbapenem-resistant infections and those with carbapenem-susceptible infections. Online databases were searched, and data were qualitatively synthesized and pooled in a metaanalysis. Nine studies met inclusion criteria: 6 retrospective case–control studies, 2 retrospective cohort studies, and 1 prospective cohort study. *Klebsiella pneumoniae* was the causative pathogen in 8 studies; bacteremia was the only infection in 5 studies. We calculated that 26%–44% of deaths in 7 studies were attributable to carbapenem resistance, and in 2 studies, which included bacteremia and other infections, −3% and −4% of deaths were attributable to carbapenem resistance. Pooled outcomes showed that the number of deaths was significantly higher in patients with carbapenem-resistant infections and that the number of deaths attributable to carbapenem resistance is considerable.

## Abstract 2

In 7 studies, rates ranged from 26% to 44%; in 2 studies, rates were −3% and −4%, respectively.

Carbapenem-resistant strains have emerged among species belonging to the *Enterobacteriaceae* family (*1*,*2*). Carbapenemases are a class of enzymes that can confer resistance to carbapenems and other β-lactam antibiotic drugs, but not all carbapenemase-producing isolates are carbapenem-resistant (*3*,*4*). Among the known carbapenemases are *Klebsiella pneumoniae* carbapenemase (KPC) and Verona integrin–encoded metallo-β-lactamase (VIM) (*5*). Several outbreaks caused by carbapenem-resistant *Enterobacteriaceae* (CRE) have been recorded in health care facilities around the world (*6*–*13*), and in some places, CRE have become endemic (*14*–*18*). Serious concurrent conditions (*3*,*4*,*19*–*22*) and prior use of fluoroquinolones (*20*,*23*,*24*), carbapenems (*22*,*25*), or broad-spectrum cephalosporins (*20*,*22*) have been independently associated with acquisition of infections caused by CRE.

Several studies have provided data regarding clinical outcomes for CRE infections. However, controversy remains concerning the number of deaths among persons infected with CRE compared with the number among persons infected with carbapenem-susceptible *Enterobacteriaceae* (CSE) (*23*,*26*). In this context, the goal of our study was to evaluate the number of deaths attributable to CRE infections by conducting a systematic review and metaanalysis of the available data.

## Methods

### Literature Search

We performed a systematic search in the PubMed (http://www.ncbi.nlm.nih.gov/pubmed/) and Scopus (http://www.scopus.com/home.url?zone=header&origin=searchbasic) databases on April 9, 2012, by using the following search terms: carbapenem-resistant or carbapenemase-producing or KPC and outcome or mortality. We also conducted a hands-on search of the reference lists of relevant studies to identify additional studies. Articles published in languages other than English, French, German, Italian, Spanish, or Greek were not evaluated.

### Study Selection Criteria

Any article that compared death rates between CRE-infected patients and CSE-infected patients was considered eligible for inclusion in the review. Studies that reported only on carbapenem-resistant isolates (without comparison with susceptible isolates) were excluded, as were studies that compared patients who had carbapenem-resistant infections with patients who were not infected. Other excluded studies were those that did not distinguish the outcomes for infected patients from those for colonized patients and studies that reported on isolates resistant to a carbapenem other than imipenem, meropenem, or doripenem. Studies that compared infection-related but not all-cause deaths among CRE-infected patients with those among CSE-infected patients were excluded because of homogeneity of the outcome analysis. Unpublished studies presented as abstracts at scientific conferences were not eligible for inclusion because of the low quantity of information provided in these types of articles.

### Data Extraction

Literature search, study selection, and data extraction were performed independently by 2 of the authors (G.S.T. and K.Z.V.). Any disagreement was resolved by consensus in meetings with all investigators and by reviewing the original articles to assess validity of the abstracted data. Extracted data included study characteristics (author, design, country, period, number of patients) and patient characteristics (type of infection, causative pathogen, and concurrent condition or severity of illness score at admission). We also recorded the all-cause deaths in each group of patients (CRE and CSE), deaths attributable to carbapenem resistance, and the independent predictors of all-cause deaths evaluated in the total population of each study.

For studies in which analyses were performed for the individual patient groups (CRE and CSE) rather than the study population as a whole, we could not conclude whether carbapenem resistance predicted death. Thus, we did not extract results from such studies.

### Definitions and Outcomes

We compared 2 groups of patients: CRE-infected and CSE-infected patients. The primary outcome of our analysis was the comparison of all-cause deaths between CRE and CSE groups with the same type of infection (i.e., bacteremia or pneumonia) caused by the same species (i.e., *K. pneumoniae*). The secondary outcome was deaths attributable to carbapenem resistance in *Enterobacteriaceae* infections. Attributable death was defined as the difference in all-cause deaths between the 2 compared groups.

Carbapenem resistance was defined as the resistance of a pathogen to imipenem, meropenem, or doripenem, according to the susceptibility breakpoints that had been applied by the investigators of each study. Carbapenemase production was not considered as carbapenem resistance if the MIC of an antibiotic was within the susceptible range according to those breakpoints.

### Statistical Analysis

We calculated pooled risk ratios (RRs) and 95% CIs regarding deaths. The statistical heterogeneity between studies was assessed by using the χ^2^ test (p<0.10 was defined to indicate the presence of heterogeneity) and the *I*^2^ index (for assessing the degree of heterogeneity) (*27*). The random effects model was applied because we considered the nonrandomized, comparative studies that we analyzed to be heterogeneous by definition. We used RevMan 5.1 software (Nordic Cochrane Centre of the Cochrane Collaboration, Copenhagen, Denmark) to perform the metaanalysis.

## Results

A total of 364 articles were retrieved during the search process: 152 in PubMed, 207 in Scopus, and 5 from hands-on searches of the reference lists of relevant studies. Of the 364 articles, 9 were considered eligible for inclusion in the analysis (*3*,*4*,*19*,*21*–*23*,*26*,*28*,*29*). The study selection process is depicted in the Figure. A total of 985 patients were included in the 9 eligible studies.

**Figure:**
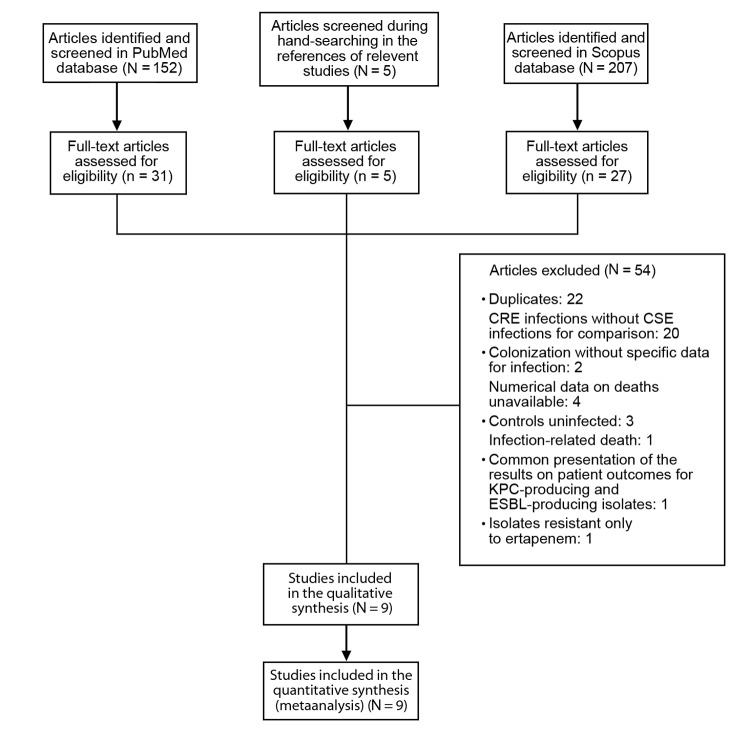
Selection process for studies included in a systematic review and metaanalysis of deaths attributable to carbapenem-resistant *Enterobacteriaceae* infections. CRE, carbapenem-resistant *Enterobacteriaceae*; CSE, carbapenem-susceptible *Enterobacteriaceae*; KPC, *Klebsiella pneumoniae* carbapenemase; ESBL, extended-spectrum β-lactamase.

The characteristics and outcomes of the included studies are presented in the Technical Appendix Table. Of the 9 studies, 8 were retrospective: 6 case-control studies with a total of 527 patients (*3*,*19*,*21*–*23*,*28*) and 2 cohort studies with a total of 296 patients (*26*,*29*). The remaining study was a prospective cohort study with 162 patients (*4*). The causative pathogen was *K*. *pneumoniae* in 8 studies (*3*,*4*,*21*–*23*,*26*,*28*,*29*) and *Escherichia coli* in 1 study (*19*). Among studies that provided relevant data, metallo-β-lactamases were the carbapenemases produced by *Enterobacteriaceae* in 2 studies (*3*,*4*), and KPC and VIM were the carbapenemases produced by *Enterobacteriaceae* in another study (*21*). In 6 studies, bacteremia represented the only infection or the majority of infections (*3*,*4*,*19*,*21*,*22*,*26*). In another study, cases of bacteremia constituted 26% of all infections (*23*). The remaining 2 studies included patients with undetermined infections (*28*) or infections other than bacteremia (*29*). An MIC of <4 μg/mL was considered the susceptibility breakpoint for imipenem, meropenem, and doripenem in 8 of the 9 studies (*3*,*4*,*19*,*21*–*23*,*26*,*29*); relevant data were not provided by 1 study (*28*).

In 3 studies, CRE-infected and CSE-infected patients had similar underlying diseases (*21*,*23*,*28*). However, in 3studies that provided specific relevant data, CRE-infected patients were more likely than CSE-infected patients to experience heart or liver failure or malignancy or to be transplant recipients (*19*,*22*,*26*). In 5 of the 9 studies, concurrent condition scores or severity of illness scores for the 2 groups of patients were compared by using the Acute Physiology and Chronic Health Evaluation II severity of disease classification system, Sequential Organ Failure Assessment scoring system, Pitt bacteremia score, or Charlson comorbidity index (*3*,*21*,*23*,*26*,*28*). Of the 5 studies, 3 showed significantly higher scores for CRE-infected than CSE-infected patients (*19*,*21*,*26*). Comparative data on the appropriateness of empirical antibiotic treatment were provided by only 2 studies (*4*,*26*). Patients with infections caused by CRE were more likely than those with infections caused by CSE to receive inappropriate empirical treatment (88% vs. 39%, odds ratio 4.1, 95% CI 1.3–12.9).

### Deaths

Reported all-cause deaths differed significantly between the 2 groups of patients in 5 of 9 studies (*3*,*4*,*19*,*22*,*26*). The CRE-attributable deaths that we calculated varied from 26% to 44% in 7 studies (*3*,*4*,*19*,*21*,*22*,*26*,*29*) and were −3% and −4%, respectively, in 2 studies (*23*,*28*).

Our pooled analysis of the 9 studies (985 patients) showed that the death rate was higher among CRE-infected than CSE-infected patients (RR 2.05, 95% CI 1.56–2.69) (Technical Appendix Figure). Moderate heterogeneity was detected between all studies (*I*^2^ = 51%). Subgroup analysis was performed for studies that included only or mostly patients with bacteremia. In 6 studies (718 patients), the patients who had bacteremia caused by CRE had higher death rates than those who had bacteremia caused by CSE (RR 2.19, 95% CI 1.82–2.63) (Technical Appendix Figure) (*3*,*4*,*19*,*21*,*22*,*26*). No heterogeneity was detected among these studies. Three studies (267 patients) provided data regarding patients with bacteremia or other infections (*23*,*28*,*29*). The death rate did not differ between CRE-infected patients and CSE-infected patients in those studies (RR 1.46, 95% CI 0.47–4.49) (Technical Appendix Figure). Considerable heterogeneity was detected among studies (*I*^2^ = 77%).

### Predictors of Death

We performed a multivariable analysis of deaths for the total study population in 7 of the 9 studies (*3*,*4*,*21*–*23*,*26*,*29*); in 6 of the 7 studies, adjustment was made for concurrent condition score or severity of illness score (*3*,*4*,*21*,*23*,*26*,*29*). In 7 studies, higher concurrent condition score at hospital admission or more severe patient condition independently predicted death (*3*,*4*,*21*–*23*,*26*,*29*). Five studies showed that carbapenem resistance was independently associated with death (*3*,*4*,*22*,*26*,*29*), and another study, which did not specifically analyze carbapenem resistance, showed that KPC production was an independent predictor of death (*21*).

## Discussion

The main finding of this metaanalysis is that the rate of CRE-attributable deaths ranged from 26% to 44% in 7 studies (*3*,*4*,*19*,*21*,*22*,*26*,*29*) and was −3% and −4%, respectively, in 2 studies (*23*,*28*). Furthermore, CRE-infected patients had an unadjusted number of deaths 2-fold higher than that for CSE-infected patients.

Six of the included studies showed significantly more deaths among CRE-infected than CSE-infected patients (*3*,*4*,*19*,*22*,*26*,*28*). In the 3 remaining studies, the lack of a significant difference in death rates for the CRE-infected and CSE-infected patients could be explained by the similarity of underlying disease characteristics for the 2 groups of patients (*21*,*23*,*28*). On the contrary, in the 3 studies that provided relevant data, concurrent condition scores or severity of illness scores were higher in CRE-infected than CSE-infected patients (*19*,*22*,*26*). In 2 studies, the Acute Physiology and Chronic Health Evaluation II score was independently associated with death (*3*,*23*).

A critical finding of our metaanalysis is that the number of deaths was 2-fold higher among patients with bacteremia caused by CRE than among patients with bacteremia caused by CSE (*3*,*4*,*19*,*21*,*22*,*26*). However, a significant difference in death rates was not detected between the 2 compared groups in studies reporting on patients with undetermined infections, patients with infections other than bacteremia, or patients among whom the percentage of bacteremia cases was low (*23*,*28*,*29*). Therefore, it could be suggested that the higher rate of death among patients with CRE infections, compared with CSE infections, is due to the higher rate of death among patients with bacteremia caused by CRE. The smaller number of patients included in this subgroup analysis (267 patients) compared with the number in the group who had bacteremia as the only infection (718 patients), along with the considerable heterogeneity among the included studies, but not among the type of infection, may justify the absence of statistical significance. Apart from the sample size, other variables that have not been analyzed might have affected the strength of the death (or outcomes) analysis. Additional and larger studies reporting on infections other than bacteremia could elucidate this issue.

Many factors other than underlying concurrent condition or severity of illness at the initial medical visit could be responsible for the higher rate of death among patients with infections caused by CRE. A key relevant factor could be the higher frequency of inappropriate empirical treatments among the CRE patients. Only 2 of the included studies provided comparative data for patients who received appropriate empirical antibiotic treatment (*4*,*26*). Those studies showed that patients with infections caused by CRE were significantly more likely than those infected by CSE to receive inappropriate antibiotic treatment. In addition, another study showed that inappropriate empirical antibiotic treatment was independently associated with death in patients infected with KPC-producing *K*. *pneumoniae* (*30*). Apart from empirical treatment, the antibiotics used for treatment might be less effective against carbapenem-resistant infections as well. There are few published clinical data available on the effectiveness of colistin, tigecycline, fosfomycin, and gentamicin (which are likely to be active in vitro against CRE) for the treatment of CSE infections. From a pharmacokinetic–pharmacodynamic perspective, these agents might be suboptimal for the treatment of serious CRE infections, particularly bloodstream infections (*31*).

Five studies showed that carbapenem resistance (*3*,*4*,*26*,*29*) or KPC production (*21*) were independent predictors of death after adjustment for concurrent condition or severity of illness. KPC ST258, a widely distributed clone of KPC-producing *K*. *pneumoniae*, is considered a successful pathogen because of its ability to persist and spread, causing nosocomial outbreaks (*32*).

Data regarding the association between carbapenem resistance and virulence are scarce. In vivo and in vitro findings from1 study argued that carbapenem-resistant *K*. *pneumoniae* isolates are less virulent and fit than carbapenem-susceptible isolates in an antibiotic-free environment (*33*).This reduction in virulence and fitness was due to the loss of the major porins OmpK35/36 (through which β-lactams penetrate into *K. pneumoniae* isolates) and the presence and expression of OmpK26 in the resistant isolates.

In addition, the number of deaths attributable to CRE infections varied between studies; the susceptibility profile of the microbes in the control groups could have an influence on this outcome. Metaanalyses have shown that death rates are higher among patients with infections caused by extended-spectrum β-lactamase–producing or multidrug-resistant *Enterobacteriaceae* isolates than among patients with infections caused by non–extended-spectrum β-lactamase or non–multidrug-resistant isolates (*34*–*36*). However, the type of infections, concurrent conditions, prior antibiotic use, and length of preinfection hospital stay could also have played a role in the observed differences in attributable death in our metaanalysis. Also, the virulence characteristics of the carbapenem-resistant isolates may differ among isolates with different types of carbapenemases or among strains that belong to different clones. This is important because some of the studies might have only included clonal isolates (e.g., KPC isolates in an endemic setting), and others might have included isolates from different clones (e.g., VIM producers that are typically polyclonal).

It should be emphasized that the findings of this systematic review and metaanalysis may not apply to the current Clinical and Laboratory Standards Institute breakpoints for carbapenem susceptibility; in 2010, the susceptibility breakpoint for imipenem, meropenem, and doripenem was lowered from 4 μg/mL to 1 μg/mL (*37*). There were no available data among the included studies that we could use to classify deaths according to the new breakpoints.

Our study findings should be interpreted in light of certain other limitations. The effect of the possible confounding factors (i.e., concurrent condition, severity of illness) on death could not be detected in the pooled analysis because only unadjusted data were entered. Furthermore, 8 of the 9 included studies had a retrospective study design. Data from such studies may be suboptimal compared with data from prospective studies, but this could not be tested due to the lack of prospective studies.

In conclusion, our findings suggest that the number of deaths attributable to carbapenem resistance is considerably high among persons with *Enterobacteriaceae* infections. Further original studies are needed to determine the reason(s) for the increased risk for death from carbapenem-resistant isolates versus carbapenem-susceptible isolates. Our findings imply a need for strict infection control measures and a need for new antibiotics to protect against CRE infections.

Technical Appendix Characteristics and outcomes of studies included in a systematic review and metaanalysis of deaths attributable to carbapenem-resistant *Enterobacteriaceae* infections and death risk ratios for patients infected with carbapenem-resistant *Enterobacteriaceae* versus carbapenem-susceptible *Enterobacteriaceae*.
