# Supplementary material for: Deaths Attributable to Carbapenem-Resistant Enterobacteriaceae Infections
Source: Emerg Infect Dis. 2014 Jul;20(7):1170–5. doi: 10.3201/eid2007.121004 (PMC4073868; doi:10.3201/eid2007.121004)
Supplement: Technical Appendix — Characteristics and outcomes of studies included in a systematic review and metaanalysis of deaths attributable to carbapenem-resistant Enterobacteriaceae infections and death risk ratios for patients infected with carbapenem-resistant Enterobacteriaceae versus carbapenem-susceptible Enterobacteriaceae. [file 12-1004-Techapp-s1.pdf]

Article DOI: http://dx.doi.org/10.3201/eid2007.121004

Deaths Attributable to Carbapenem-Resistant *Enterobacteriaceae* Infections

Technical Appendix

Technical Appendix Table. Characteristics and outcomes of studies included in a systematic review and metaanalysis of deaths attributable to carbapenem-resistant *Enterobacteriaceae* infections*

Study first author, year (reference)	Study design, year(s), country	No. patients, characteristic	Type of infection	Concurrent condition or severity of disease score on admission (CRE vs. CSE)	Type/location of death reported	Deaths		Attributable deaths (%)†	Independent predictors of death‡
						No. CRE-associated/ no. total (%)	No. CSE-associated/ no. total (%)		
Ben-David, 2012 (*1*)	Retrospective cohort, 2006, Israel	192	BSI	Pitt bacteremia score, median: 4 vs. 2 (CSE) and 3 (ESBL-producing)§	In hospital	29/42 (69)	45/150 (30)	39	Carbapenem resistance, Charlson comorbidity index, Pitt bacteremia score
Chang, 2011 (*2*)	Retrospective matched (1:1 by sex, age, year of hospital admission, LOS up to the isolation of *Escherichia coli*) case-control, 2006–2008, Taiwan	51	BSI	Pitt bacteremia score, mean ± SD: 5.6 ± 4.4 vs. 2.5 ± 2.6	In hospital	16/17 (94)	17/34 (50)	44	NR
Gaviria, 2011 (*3*)	Retrospective matched (1:1 by age, date of specimen collection) case-control, 2009–2011, USA	57	Undetermined infections	Charlson comorbidity index score, median: 2 vs. 1.1	NR	1/19 (5)	3/38 (8)	−3	NR
Mouloudi, 2010 (*4*)	Retrospective nested case-control study, 2007–2008, Greece	59, ICU patients	BSI	APACHE II score, median: 26 (KPC-producing) and 20 (MBL-producing) vs. 17.5	In hospital	25/37 (68)	9/22 (41)	27	KPC-production, SOFA score, solid organ transplantation, age
Daikos, 2009 (*5*)	Multicenter prospective cohort, 2004–2006, Greece	162, 33% ICU patients	BSI	NR	All-cause 14-d	6/14 (43)	25/148 (17)	26	MIC of carbapenems >4 μg/mL, age, rapidly fatal underlying disease (McCabe and Jackson classification). After adjustment for inappropriate empirical or definitive antibiotic treatment: age, rapidly fatal underlying disease

Study first author, year (reference)	Study design, year(s), country	No. patients, characteristic	Type of infection	Concurrent condition or severity of disease score on admission (CRE vs. CSE)	Type/location of death reported	Deaths: No. CRE-associated/ no. total (%)	Deaths: No. CSE-associated/ no. total (%)	Attributable deaths (%)†	Independent predictors of death‡
Patel, 2008 (*6*)	Retrospective matched (1:1 by site of infection) case-control, 2004–2006, USA	198	72% BSI (primary and secondary), 34% intra-abdominal infection, 2% urosepsis, 2% ventriculitis, 2% osteomyelitis, 2% empyema, 1% deep sinus infection	NR	In hospital	48/99 (48)	20/99 (20)	28	Heart disease, liver disease, ICU admission, carbapenem resistance
Schwaber, 2008 (*7*)	Retrospective cohort, 2003–2006, Israel	104	Various infections#	NR	In hospital	21/48 (44)	7/56 (13)	31	Carbapenem resistance, malignancy, mechanical ventilation
Daikos, 2007 (*8*)	2-center retrospective case-control, 2003–2004, Greece	56, 46% ICU-patients	BSI	NR	All-cause 14-d	7/13 (54)	5/43 (12)	42	Imipenem resistance, APACHE II score
Falagas, 2007 (*9*)	2-center retrospective matched (1:1 by site of infection) case-control, 2000–2006, Greece	106, 60% ICU patients	26% BSI, 23% pneumonia, 23% UTIs, 15% surgical site infection, 8% catheter-related infection, 6% genital tract infections	APACHE II score, mean ± SD: 14.4 ± 8.8 vs. 12.4 ± 6.1	In hospital	16/53 (30)	18/53 (34)	−4	APACHE II score

*Except for the causative pathogen (*Escherichia coli*) in the 2011 study by Chang et al., the causative pathogen in all studies was *Klebsiella pneumoniae*. CRE, carbapenem-resistant *Enterobacteriaceae*; CSE, carbapenem-susceptible *Enterobacteriaceae*; BSI, bloodstream infection; ESBL, extended-spectrum β-lactamase; LOS,: length of stay; NR, not reported; ICU, intensive care unit; APACHE, Acute Physiology and Chronic Health Evaluation; KPC, *K. pneumoniae* carbapenemase; MBL, metallo-β-lactamase; SOFA, Sequential Organ Failure Assessment; UTIs, urinary tract infections
†Attributable death was calculated by the authors of this review as the difference in all-cause death between the 2 compared groups.
‡Only the results of multivariable analyses reported on the total study population and not on each patient group (CRE and CSE) separately were extracted.
§ESBL-producing bacteria were considered as carbapenem-susceptible.
#Carbapenem-resistant group: 40% UTIs, 19% wound/skin/soft tissue infections, 13% blood/intravenous line–related infections; carbapenem-susceptible group: 55% UTIs, 15% wound/skin/soft tissue infections, 15% blood/intravenous line–related infections

Study or Subgroup	Carba-resistant Events	Total	Carba-susceptible Events	Total	Weight	Risk Ratio M-H, Random, 95% CI
**1.1.1 Bacteremia**						
Ben-David 2012	29	42	45	150	18.4%	2.30 [1.68, 3.16]
Chang 2011	16	17	17	34	17.3%	1.88 [1.32, 2.69]
Daikos 2007	7	13	5	43	6.0%	4.63 [1.76, 12.16]
Daikos 2009	6	14	25	148	9.3%	2.54 [1.26, 5.12]
Mouloudi 2010	25	37	9	22	12.2%	1.65 [0.95, 2.86]
Patel 2008	48	99	20	99	14.9%	2.40 [1.54, 3.73]
**Subtotal (95% CI)**		**222**		**496**	**78.1%**	**2.19 [1.82, 2.63]**
Total events	131		121			
Heterogeneity: Tau² = 0.00; Chi² = 4.53, df = 5 (P = 0.48); I² = 0%						
Test for overall effect: Z = 8.30 (P < 0.00001)						
**1.1.2 Bacteremia or other infections**						
Falagas 2007	16	53	18	53	12.1%	0.89 [0.51, 1.55]
Gaviria 2011	1	19	3	38	1.4%	0.67 [0.07, 5.99]
Schwaber 2008	21	48	7	56	8.4%	3.50 [1.63, 7.51]
**Subtotal (95% CI)**		**120**		**147**	**21.9%**	**1.46 [0.47, 4.49]**
Total events	38		28			
Heterogeneity: Tau² = 0.68; Chi² = 8.68, df = 2 (P = 0.01); I² = 77%						
Test for overall effect: Z = 0.65 (P = 0.51)						
**Total (95% CI)**		**342**		**643**	**100.0%**	**2.05 [1.56, 2.69]**
Total events	169		149			
Heterogeneity: Tau² = 0.08; Chi² = 16.49, df = 8 (P = 0.04); I² = 51%						
Test for overall effect: Z = 5.17 (P < 0.00001)						
Test for subgroup differences: Chi² = 0.49, df = 1 (P = 0.49), I² = 0%						

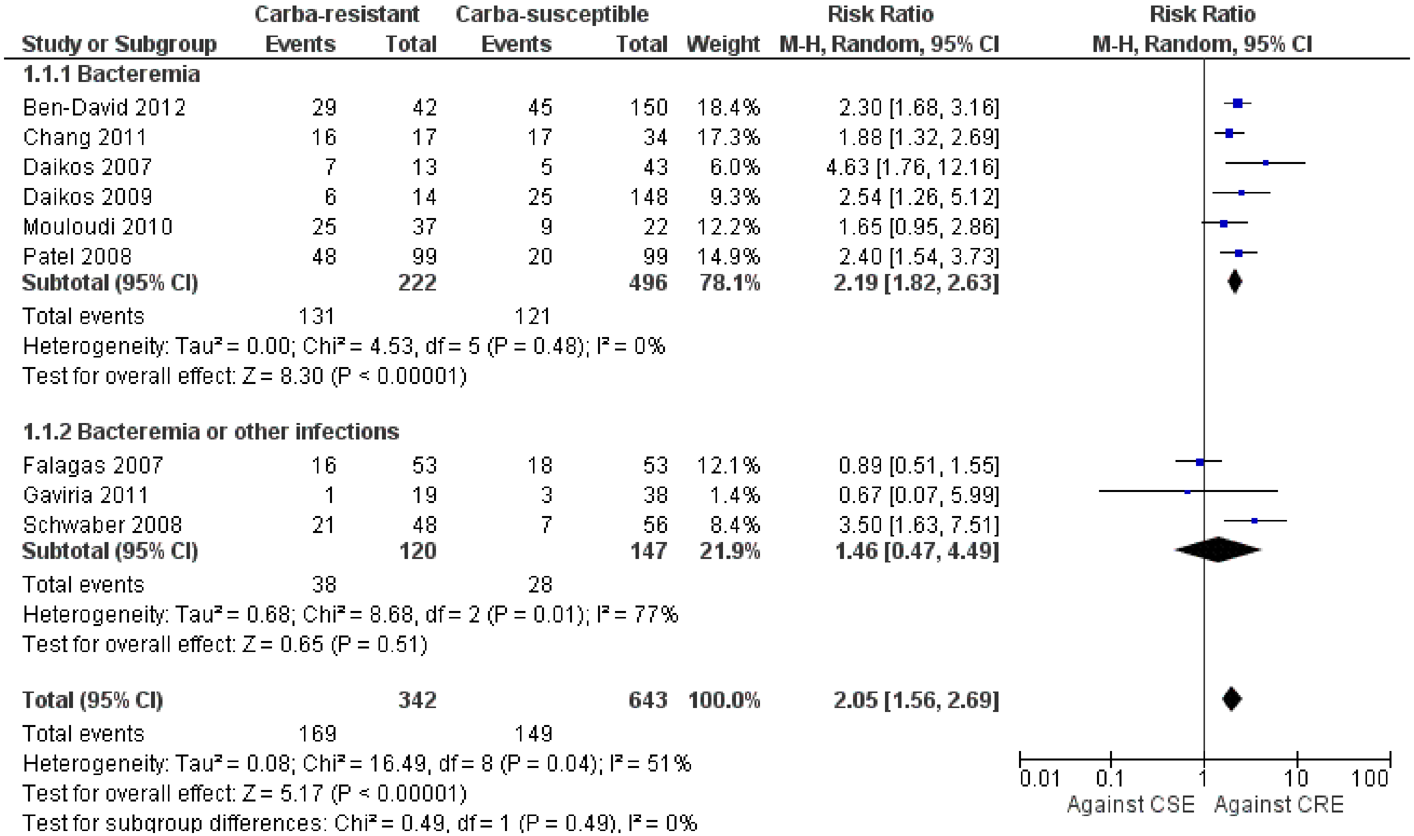

Technical Appendix Figure. Death risk ratios (RRs) for patients infected with carbapenem-resistant Enterobacteriaceae (CRE) versus carbapenem-susceptible *Enterobacteriaceae* (CSE). Vertical line represents the point of no difference between carbapenem-resistant and carbapenem-susceptible pathogens; squares represent RRs; diamonds represent pooled RRs for all studies; horizontal lines represent 95% CIs. RRs were determined by using a Mantel-Haenszel (M-H) random effects model. Carba-resistant, CRE; Carba-susceptible, CSE. References: Ben-David 2012 (*1*); Chang 2011 (*2*); Daikos 2007 (*8*); Daikos 2009 (*5*); Mouloudi 2010 (*4*); Patel 2008 (*6*); Falagas 2007 (*9*); Gaviria 2011 (*3*); Schwaber 2008 (*7*).

References

1. Ben-David D, Kordevani R, Keller N, Tal I, Marzel A, Gal-Mor O, et al. Outcome of carbapenem resistant *Klebsiella pneumoniae* bloodstream infections. Clin Microbiol Infect. 2012;18:54–60. PubMed http://dx.doi.org/10.1111/j.1469-0691.2011.03478.x

2. Chang HJ, Hsu PC, Yang CC, Kuo AJ, Chia JH, Wu TL, et al. Risk factors and outcomes of carbapenem-nonsusceptible *Escherichia coli* bacteremia: a matched case-control study. J Microbiol Immunol Infect. 2011;44:125–30. PubMed http://dx.doi.org/10.1016/j.jmii.2010.06.001

3. Gaviria D, Bixler D, Thomas CA, Ibrahim SM, Kallen A, Limbago B, et al. Carbapenem-resistant *Klebsiella pneumoniae* associated with a long-term–care facility—West Virginia, 2009–2011. MMWR Morb Mortal Wkly Rep. 2011;60:1418–20. PubMed

4. Mouloudi E, Protonotariou E, Zagorianou A, Iosifidis E, Karapanagiotou A, Giasnetsova T, et al. Bloodstream infections caused by metallo-beta-lactamase/*Klebsiella pneumoniae* carbapenemase–producing *K. pneumoniae* among intensive care unit patients in Greece: risk factors for infection and impact of type of resistance on outcomes. Infect Control Hosp Epidemiol. 2010;31:1250–6. PubMed http://dx.doi.org/10.1086/657135

5. Daikos GL, Petrikkos P, Psichogiou M, Kosmidis C, Vryonis E, Skoutelis A, et al. Prospective observational study of the impact of VIM-1 metallo-beta-lactamase on the outcome of patients with *Klebsiella pneumoniae* bloodstream infections. Antimicrob Agents Chemother. 2009;53:1868–73. PubMed http://dx.doi.org/10.1128/AAC.00782-08

6. Patel G, Huprikar S, Factor SH, Jenkins SG, Calfee DP. Outcomes of carbapenem-resistant *Klebsiella pneumoniae* infection and the impact of antimicrobial and adjunctive therapies. Infect Control Hosp Epidemiol. 2008;29:1099–106. PubMed http://dx.doi.org/10.1086/592412

7. Schwaber MJ, Klarfeld-Lidji S, Navon-Venezia S, Schwartz D, Leavitt A, Carmeli Y. Predictors of carbapenem-resistant *Klebsiella pneumoniae* acquisition among hospitalized adults and effect of acquisition on mortality. Antimicrob Agents Chemother. 2008;52:1028–33. PubMed http://dx.doi.org/10.1128/AAC.01020-07

8. Daikos GL, Karabinis A, Paramythiotou E, Syriopoulou VP, Kosmidis C, Avlami A, et al. VIM-1–producing *Klebsiella pneumoniae* bloodstream infections: analysis of 28 cases. Int J Antimicrob Agents. 2007;29:471–3. PubMed http://dx.doi.org/10.1016/j.ijantimicag.2006.11.006

9. Falagas ME, Rafailidis PI, Kofteridis D, Virtzili S, Chelvatzoglou FC, Papaioannou V, et al. Risk factors of carbapenem-resistant *Klebsiella pneumoniae* infections: a matched case control study. J Antimicrob Chemother. 2007;60:1124–30. PubMed http://dx.doi.org/10.1093/jac/dkm356
